# *spa* diversity of methicillin-resistant and -susceptible *Staphylococcus aureus* in clinical strains from Malaysia: a high prevalence of invasive European *spa*-type t032

**DOI:** 10.7717/peerj.11195

**Published:** 2021-04-08

**Authors:** Sherry Usun Jones, Kek Heng Chua, Ching Hoong Chew, Chew Chieng Yeo, Fatimah Haslina Abdullah, Norlela Othman, Boon Pin Kee, Suat Moi Puah

**Affiliations:** 1 Department of Biomedical Science, Faculty of Medicine, University of Malaya, Kuala Lumpur, Malaysia; 2 Faculty of Health Sciences, Universiti Sultan Zainal Abidin, Kuala Nerus, Terengganu, Malaysia; 3 Faculty of Medicine, Universiti Sultan Zainal Abidin, Kuala Terengganu, Terengganu, Malaysia; 4 Department of Pathology, Hospital Sultanah Nur Zahirah, Kuala Terengganu, Terengganu, Malaysia

**Keywords:** Methicillin-resistant *Staphylococcus aureus*, Methicillin-susceptible *Staphylococcus aureus*, *spa* typing, t032

## Abstract

**Background:**

* Staphylococcus aureus* is one of the important pathogens causing nosocomial infection. *spa* typing allows identification of *S. aureus* clones in hospital isolates and is useful for epidemiological studies and nosocomial infection control. This study aims to investigate the *spa* types in Malaysian *S. aureus* isolates obtained from various clinical specimens.

**Method:**

A total of 89 methicillin-resistant *S. aureus* (MRSA) [pus (*n* = 55), blood (*n* = 27), respiratory (*n* = 5), eye (*n* = 2)] isolates and 109 methicillin-susceptible *S. aureus* (MSSA) [pus (*n* = 79), blood (*n* = 24), respiratory (*n* = 3), eye (*n* = 2) and urine (*n* = 1)] isolates were subjected to *spa* typing with sequences analysed using BioNumerics version 7.

**Results:**

The *spa* sequence was successfully amplified from 77.8% of the strains (154/198) and 47 known *spa* types were detected. The distribution of known *spa* types in MRSA (36.2%, 17/47) was less diverse than in MSSA (70.2%, 33/47). The most predominant *spa* types were t032 (50%) in MRSA, and t127 (19%) and t091 (16.7%) in MSSA, respectively. *spa* type t091 in MSSA was significantly associated with skin and soft tissue infections (*p* = 0.0199).

**Conclusion:**

The previously uncommon *spa* type t032 was detected in the Malaysian MRSA strains, which also corresponded to the most common *spa* type in Europe and Australia, and has replaced the dominant *spa* type t037 which was reported in Malaysia in 2010.

## Introduction

*Staphylococcus aureus* is both a commensal bacterium and human pathogen that has potential to cause a wide variety of infections, ranging from bacteraemia, infective endocarditis, osteoarticular, skin and soft tissue, pleuropulmonary and device-related infections (Jokinen et al., 2018). The first emergence of methicillin-resistant *S. aureus* (MRSA) was associated with hospital-acquired infections (HA-MRSA) in the early 1960s, but it has spread to the community and was referred to as community-acquired MRSA (CA-MRSA) (Turner et al., 2019). Generally, HA-MRSA and CA-MRSA are differentiated by functional genomic traits (Otto, 2013).

Methicillin-resistant *S. aureus* has been known to evolve from methicillin-susceptible *Staphylococcus aureus* (MSSA) due to the acquisition of *mecA* gene, which encodes the low-affinity penicillin-binding protein 2a (PBP2a) via horizontal transfer located in the mobile genetic element known as staphylococcal cassette chromosome *mec* (*SCCmec*). This mechanism allows the bacteria to become resistant to a wide range of *β*-lactam antibiotics (Kirmusaoglu, 2018; Wu et al., 2019). However, in 2007, a novel gene showed 69% sequence identity to the original *mecA* gene, which is now termed as *mecC*. The *mecC* gene has also shown resistance towards oxacillin and cefoxitin (Lakhundi & Zhang, 2018). Overall, the number of antibiotics that are effective against MRSA is declining as the years passed which inclined to a future where antibiotics are less effective (Mulani et al., 2019). Hence, MRSA is listed as one of the World Health Organization’s high priority pathogens for which new and effective antibiotics are urgently needed.

MRSA infections has caused persistently high mortality across the world. The prevalence rates of MRSA in hospitals in Asian countries such as China, South Korea, Japan and Taiwan ranged from 70 to 80% (Wu et al., 2019; Ghaznavi-Rad et al., 2010). The frequency of MRSA resistance toward the older line of antibiotics such as erythromycin, gentamicin and ciprofloxacin showed a fluctuating trend from 1990 to 2017 in Malaysia but the overall prevalence of antibiotic resistance among MRSA remained above 70%. On the contrary, the prevalence of antibiotic resistance among MSSA strains were much lower below 18% for all seven antibiotics tested—-erythromycin, gentamicin, ciprofloxacin, co-trimoxazole, clindamycin, rifampin and fusidic acid (McNeil et al., 2016; Che Hamzah et al, 2019a).

According to the Malaysian National Antibiotic Resistance Surveillance Report from 2004 to 2017, the prevalence rate of MRSA among *S. aureus* clinical isolates in the country ranged from 17.2% to 28.1% (Ministry of Health Malaysia, 2017). Most published studies of isolates from Malaysia focused on the spread of MRSA in hospitals and the community rather than MSSA, thus data concerning Malaysian MSSA infections is limited (Che Hamzah et al., 2019a).

Prior to the emergence of MRSA, MSSA was the prominent cause of both serious and uncomplicated *S. aureus* infections. Some cases describing outbreaks and global spread of MSSA in hospitals affecting neonatal units and hospital staff in UK, USA and Canada were reported, and today this organism still remains as a prime species in hospital infections (DeLeo et al., 2011; Uhlemann et al., 2014; Monaco et al., 2017). Therefore, the understanding of population structure of MRSA and MSSA is important and this can be achieved by identifying the dominant strains circulating in the hospital settings associated with disease outbreaks. The result also helps to monitor bacterial resistance, transmission chain and provide insight into clinical infection controls.

There are various molecular typing tools to characterize the population structure of *S. aureus* such as pulsed field gel electrophoresis, multilocus sequence typing, SCC*mec* typing and *spa* typing (Lakhundi & Zhang, 2018). *spa* typing is a reliable and discriminative method based on the sequencing analysis of variable number tandem repeats in the highly polymorphic region X of the *spa* gene encoding for staphylococcal protein A (SpA). The SpA is a surface protein contributing to *S. aureus* pathogenesis by binding to immunoglobulin which allows the bacteria to be inaccessible to opsonins hence impairing phagocytosis (Asadollahi et al., 2018). The *spa* region consists of variable number of 21- to 27-bp repeats (Mun & Hwang, 2019). The *spa* type distribution of *S. aureus* isolates exhibits different patterns in different geographic location around the world. Constant emergence of new strains that are continuously evolving over time often results in sustained epidemics (Lakhundi & Zhang, 2018). Some studies have suggested that certain *S. aureus* lineages exhibited a significant trend toward hematogenous complications (Fowler et al., 2017; Rasmussen et al., 2013).

The distribution of *spa* types in Asia is well studied; however, detailed report on the distribution of *spa* type of MRSA and MSSA strains from clinical isolates in Malaysia remains scanty. Most studies reported *spa* type t037 being the predominant type in Malaysia collected from clinical sources, though some studies also reported from the community (Wu et al., 2019; Ghasemzadeh-Moghaddam et al., 2011; Zarizal et al., 2018). The aims of the present study were: (i) to investigate the distribution of *spa* types in MRSA and MSSA strains collected from clinical sources from a tertiary hospital in the state of Terengganu, Malaysia and (ii) to assess the potential association between the *spa* type and clinical presentation.

## Material and Methods

### Bacterial strains

A total of 198 bacterial strains which consisted of 89 clinical MRSA [pus (*n* = 55), blood (*n* = 27), respiratory (*n* = 5), eye (*n* = 2)] and 109 clinical MSSA [pus (*n* = 79), blood (*n* = 24), respiratory (*n* = 3), eye (*n* = 2) and urine (*n* = 1)] isolates that were obtained from Hospital Sultanah Nur Zahirah, Terengganu from July 2016 to June 2017 in a previous study (Che Hamzah et al., 2019b) and that had been cryopreserved in 20% glycerol at −80 °C were used. *S. aureus* isolates were grown in Luria-Bertani (LB) broth overnight at 37 °C.

### Ethics statement

The samples obtained are mainly used for diagnostic laboratory testing in the Hospital Sultanah Nur Zahirah, Terengganu and sample collection were approved by the Malaysia Ministry of Health, Medical Research and Ethics Committee with National Medical Research Registry no: NMRR-15-2369-28130 (IIR).

### DNA extraction

Total genomic DNA was extracted from overnight cultures using in-house boiling method (Puah et al., 2018). Briefly, one mL of bacterial culture was harvested by centrifugation at 13,000 rpm for 10 min and was washed twice with distilled water. A total of 100 µL sterile distilled water was added to resuspend the pellet and followed by boiling at 95 °C for 10 min. The cell lysate was incubated on ice for 10 min and pelleted down, followed by transferring the supernatant to a new microcentrifuge tube. The quantity and quality of the total genomic DNA was measured spectrophotometrically. The extracted DNA was stored at −20 °C for further investigation.

### *spa* typing

The *spa* locus was PCR-amplified using the *spa* primer pair, *spa*-1113F (5′-TAAAGACGATCCTTCGGTGAGC-3) and *spa*-1618R (5′-TTAGCATCTGCATGGTTTGC-3), as described previously (Larsen, Stegger & Sorum, 2008). DNA amplification was carried out in 25 µL PCR mixture consists of 2.5 µL of 10×  Dream*Taq* PCR buffer (20 mM Tris HCl, pH 8.0, 1 mM DTT, 0.1 mM EDTA, 100 mM KCl, 0.5% Nonidet P40, 0.5% Tween 20 and 50% glycerol), 0.4 µM of each 1113F and 1618R primers, 1 U of Dream*Taq* DNA polymerase (Thermo Fisher, USA), 0.2 mM of each dNTPs and 25 ng of DNA template. The thermal cycling condition was set at initial denaturation at 95 °C for 3 min, followed by 30 cycles of 95 °C for 1 min, 52 °C for 45 s and 72 °C for 45 s, with a final extension at 72 °C for 10 min. The amplified products were visualised on 1% (w/v) agarose gel, followed by purification and subjected to DNA sequencing in a commercial sequencing facility (Apical Laboratories, Malaysia). *spa* types were subsequently assigned using BioNumerics version 7 (Applied Math, Belgium).

### Data analysis

Analysis of *spa* sequences was performed using the *spa* typing plug-in tool of the BioNumerics version 7 (Applied Maths, Belgium) via DSI (duplication, substitutions and indels) model for pairwise alignment of repeats. With this plug-in, a similarity matrix was generated based on the DSI model and used to construct a minimum spanning tree (MST). MST was constructed with the node distance between *spa* types using Dice correction, the node size of *spa* frequency and a bin unit distance that was set to 1.0. Therefore, the distance between *spa* types of 99% to 100% similarity was 0 bin distance whereas 98% to 99% similarity was assigned a distance of 1, on MST. In the analysis of association of *spa* types and clinical characteristics, data was analysed using Fisher’s exact test with a 2 × 2 contingency table and differences at *p* < 0.05 calculated with two tails was considered statistically significant (McDonald, 2014).

## Results

### Distribution of *spa* type

Overall, the *spa* locus was successfully amplified from 77.8% (154/198) of the strains. Of the forty-four non-typeable strains (i.e., in which no *spa* amplified product was obtained), 19 were MRSA and 25 were MSSA. The nucleotide sequence analysis of the 154 typeable strains revealed 47 distinct *spa* types. Distribution of the most common (n ≥ 2) *spa* types in MRSA and MSSA strains according to clinical source is presented in Table 1. The distribution of *spa* types in MRSA was less diverse than in MSSA as 36.2% (17/47) were observed among MRSA whereas 70.2% (33/47) were found in MSSA strains. Of the 33 *spa* types in the MSSA population, 90.9% (30/33) were only detected once in the MSSA population. Three *spa* types were detected in both MRSA and MSSA, i.e., *spa* type t127 (3 MRSA, 16 MSSA), *spa* type t315 (1 MRSA, 5 MSSA) and *spa* type t548 (2 MRSA, 2 MSSA), while all the other *spa* types were found only either in MRSA or in MSSA strains. The majority of the MRSA strains belonged to t032 (50%, 35/70), followed by t304 (11.4%, 8/70), and t022 (7.1%, 5/70). The majority of MSSA strains belonged to t127 (19%, 16/84), followed by t091 (16.7%, 14/84), t189 (8.3%, 7/84) and t084 (8.3%, 7/84).

**Table 1:** Distribution of *spa* type (*n* ≥ 2) and sequence type among MRSA and MSSA isolates.

	***Spa* type**	**No. of strains (*n*, %)**						**ST**
		**Blood**	**Pus**	**Respiratory**	**Eye**	**Urine**	**Total**	
**MRSA**	t032	12 (13.4)	18 (20.2)	4 (4.4)	1 (1.1)	–	35 (39.3)	ST-22
	t304	1 (1.1)	7 (7.8)	–	–	–	8 (9.0)	ST-6
	t022	–	4 (4.4)	1 (1.1)	–	–	5 (5.6)	ST-22
	t127	1 (1.1)	2 (2.2)	–	–	–	3 (3.4)	ST-1
	t458	3 (3.3)	–	–	–	–	3 (3.4)	ST-225
	t037	2 (2.2)	1 (1.1)	–	–	–	3 (3.4)	ST-239
	t548	–	1 (1.1)	–	1 (1.1)	–	2 (2.2)	ST-5/97
	t3841	1 (1.1)	1 (1.1)	–	–	–	2 (2.2)	ST-672
**MSSA**	t127	4 (3.6)	11 (10.1)	1 (0.9)	–	–	16 (14.7)	ST-1
	t091	1 (0.9)	12 (11.0)	–	–	1 (0.9)	14 (12.8)	ST-7
	t189	1 (0.9)	6 (5.5)	–	–	–	7 (6.4)	ST-188
	t084	3 (2.7)	3 (2.7)	–	1 (0.9)	–	7 (6.4)	ST-15/18
	t315	2 (1.8)	3 (2.7)	–	–	–	5 (4.6)	ST-361
	t003	–	3 (2.7)	–	–	–	3 (2.8)	ST-5/225
	t2078	1 (0.9)	2 (1.8)	–	–	–	3 (2.8)	ST-101
	t548	1 (0.9)	1 (0.9)	–	–	–	2 (1.8)	ST-5/97
	t2526	–	2 (1.8)	–	–	–	2 (1.8)	ST-88
	t4407	1 (0.9)	1 (0.9)	–	–	–	2 (1.8)	NA

**Notes.**

MRSA Methicillin-resistant *Staphylococcus aureus* MSSA Methicillin-sensitive *Staphylococcus aureus* ST Sequence type NA Not available

### Genetic diversity of *spa* types

**Figure 1:**
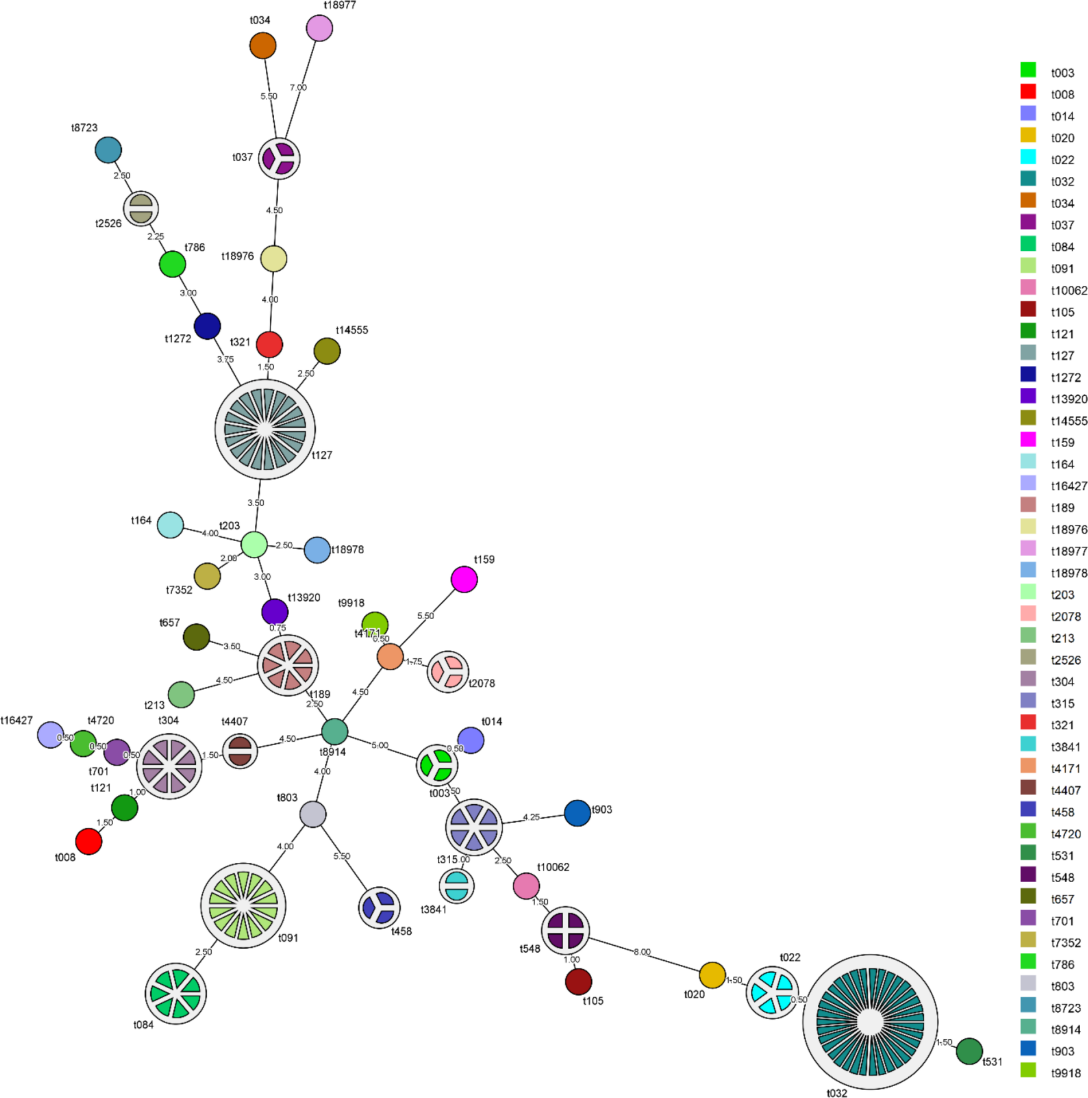
Minimum spanning tree (MST) of the clinical MRSA and MSSA strains. MST was performed using the *spa* typing plug-in tool of the BioNumerics software version 7. The node distance between *spa* type was computed using Dice correction. Each *spa* type is depicted by a single node, node size is proportional to *spa* frequency and line length is proportional to the number of mutational steps between the types. A bin distance of 1.0 was used for the MST parameter.

The MST showing the relationship among *spa* types detected in the Malaysian *S. aureus* strains was shown in Fig. 1. *spa* type t022 which accounts for 7.1% (5/70) of MRSA strains was closely related to the predominant *spa* type t032 in MRSA (50%, 35/70) with 98.5% similarity. Repeats among the known *spa* types varied from one repeat (t458) to sixteen repeats (t032). The distribution of known *spa* types with repeat succession above 95% similarity is presented in Table 2. *spa* type t022 and t032 shared similar sequences (98.5%) except for a single variation due to deletion of one repeat unit in a repeat sequence (t022: 26-23-13-23-31-29-17-31-29-17-25-17-25-16-28 and t032: 26-23-**23**-13-23-31-29-17-31-29-17-25-17-25-16-28). Another *spa* type, t084 (07-23-12-34-34-12-12-23-02-12-23) which accounts for 8.3% (7/84) of MSSA strains, was closely related genetically to the most common *spa* type in MSSA strains, *spa* type t091 (07-23-21-17-34-12-23-02-12-23) (16.7%, 14/84) with 96.5% similarity.

**Table 2:** Distribution of *spa* type with repeat succession above 95% similarity in sources of isolation.

***Spa* type**	**Repeat succession**																**Strains from clinical source, *n***					
																	Pus	Blood	Respiratory	Urine	Eye	Total
**t022**	26	23		13	23	31	29	17	31	29	17	25	17	25	16	28	4		1			5
**t032**	26	23	23	13	23	31	29	17	31	29	17	25	17	25	16	28	18	12	4		1	35
**t084**	07	23			12	34	34	12	12	23	02	12	23				3	3			1	7
**t091**	07	23	21	17			34		12	23	02	12	23				12	1		1		14
**t127**	07	23	21	16	34	33	13										11	4	1			16
**t321**	07	23		16	34	33	13										1					1

### Association of *spa* types and clinical manifestations caused by MRSA and MSSA

Analysis on the association of *spa* type and the clinical manifestations caused by MRSA and MSSA strains is shown in Table 2. The predominant s*pa* type t032 and t091 in this study were more commonly associated with skin and soft tissue infections (SSTIs) with 36% (9/25) and 25% (12/48) respectively. However, only *spa* type t091 from MSSA strains was significantly associated with SSTI (*p* = 0.0199) (Table 3).

**Table 3:** Association of *spa* type and clinical manifestations caused by MRSA and MSSA.

		*Spa* type	**SSTI**, **n (%)**	**Non-SSTI, n (%)**									***P* value**
				Total	B	P	PD	OA	IOA	IE	OM	Others	
**MRSA**		t032	9 (36)	26 (58)	5 (11)	5 (11)	2 (4)	1 (2)	3 (7)	1 (2)		9 (20)	0.1338
		t304	4 (16)	4 (9)	1 (2)		1 (2)	2 (4)					0.4435
		t022	3 (12)	2 (4)								2 (4)	0.3405
		t127	0 (0)	3 (7)	1 (2)	1 (2)	1 (2)						0.5473
		t458	1 (4)	2 (4)	2 (4)								1.0000
		t037	1 (4)	2 (4)	1 (2)		1 (2)						1.0000
		t548	1 (4)	1 (2)	1 (2)								1.0000
		t3841	2 (8)	0 (0)									0.1242
		Others	4 (16)	5 (11)									0.7119
		Total	25	45									
**MSSA**		t127	7 (15)	9 (25)	2 (6)	1 (3)	1 (3)				1 (3)	4 (11)	0.2692
		t091	12 (25)	2 (6)		1 (3)						1 (3)	0.0199^*^
		t189	4 (8)	3 (8)		1 (3)	1 (3)					1 (3)	1.0000
		t084	3 (6)	4 (11)	3 (8)	1 (3)							0.4551
		t315	2 (4)	3 (8)	2 (6)	1 (3)							0.6470
		t003	2 (4)	1 (3)		1 (3)							1.0000
		t2078	1 (2)	2 (6)								2 (6)	0.5738
		t548	1 (2)	1 (3)	1 (3)								1.0000
		t2526	2 (4)	0 (0)									0.5043
		t4407	2 (4)	0 (0)									0.5043
		Others	12 (25)	11 (31)									0.0634
		Total	48	36									

**Notes.**

Others in MRSA; t315, t203, t657, t020, t8723, t803, t531, t10062, t18978.

Others in MSSA; t008, t786, t213, t903, t014, t164, t8914, t4171, t9918, t321, t701, t1272, t034, t121, t7352, t105, t4720, t159, t18977.

SSTI skin and soft tissue infection B bacteraemia P pleuropulmonary PD prosthetic device OA osteoarticular IOA internal organ abscess IE infective endocarditis OM otitis media

others; epidural abscess, meningitis, toxic shock syndrome, urinary tract infection.

* *p* value <0.05.

## Discussion

Overall, the *spa* gene was detected in 154 (70 MRSA, 84 MSSA) out of 198 clinical strains, but not in the remaining 44 strains (19 MRSA, 25 MSSA), which were thus referred as non-typeable. This may due to either mutations in the *spa* gene which prevented the *spa* primers from annealing and hence, unable to be PCR-amplified, or a true deficiency of *spa*. Baum et al. (2009) were the first to describe the occurrence of natural protein A mutations in two non-*spa* typeable clinical *S. aureus* strains. Both strains were isolated from blood cultures of patients with severe *S. aureus* infections in Denmark, indicating that the strains were still virulent and invasive in spite of being *spa*-deficient. On the other hand, Brignoli and co-workers (2019) reported that two MSSA and seven MRSA strains did not express *spa* in a collection of 133 staphylococcal strains due to the presence of mutations in the *spa* gene. Another possible reason is due to the weakness of commonly used *spa* typing primers as rearrangements in the IgG-binding region of the *spa* gene lead to 1–2% of strains to be designated as “non-typeable”  (Votintseva et al., 2014).

The most common *spa* type, t032, was also the most prevalent *spa* type causing invasive MRSA infections in Europe. This *spa* type is reported as one of the *spa* types associated with the epidemic MRSA known as EMRSA-15 which emerged in the 1980s and spread rapidly in the late 1990s to become the dominant clone of hospital acquired-MRSA strains in the United Kingdom (Grundmann et al., 2010). Interestingly, this clone is reportedly rare outside of European countries. However, it was first detected at two different hospitals in Malaysia, i.e., Hospital Kuala Lumpur and University of Malaya Medical Centre, in 2007 (Wu et al., 2019; Lim et al., 2012). This is the first report which described the dominance of *spa* type t032 (50%, 35/70) in Malaysia. Our findings showed that *spa* type t032 colonizes and establishes infection in a wide range of body sites suggesting that it does not possess a tropism for specific sites of infections. Nevertheless, this *spa* type has caused four deaths isolated from blood culture related to sepsis (2), septic shock (1), and hepatic encephalopathy and coagulopathy (1) as well as three deaths isolated from respiratory specimens from one infant and two adults admitted to the intensive care unit.

A single base pair change within the *spa* gene can produce different *spa* type but still highly related (Durand et al., 2018). For example, *spa* type t022 is a shorter form of *spa* type t032 with the deletion of one repeat and was detected in this study. *spa* type t022 is one of the most frequent *spa* types found in Europe and associated with EMRSA-15, however, it has never been reported in Malaysia (Asadollahi et al., 2018; Grundmann et al., 2010). In this study, the frequency of *spa* type t022 was low, accounting for 7.1%, (5/70) among MRSA strains but it still caused the death of a 41-year old patient from cellulitis and pneumonia.

In most Asian countries, *spa* type t037 was the dominant MRSA strain found in clinical settings except for South Korea and Japan (Wu et al., 2019). In Malaysia, *spa* type t037 was first detected in 2003 (Lim et al., 2012). Over 90% of MRSA strains isolated from 2007 and 2008 belonged to *spa* type t037 (Lim et al., 2013; Wu et al., 2019). However, the *spa* type t037 in the present study accounted for only 4.3% (3/70) of all MRSA strains. The *spa* type t037, also known as the Hungarian/Brazilian clone, was one of the most successful MRSA lineages that is now being replaced by the *spa* type t032 (Holden et al., 2013; Knight et al., 2012). The second most common *spa* type in this study, *spa* type t304, which accounted for 11.4% of our MRSA strains was first detected in 2007 by  Lim et al. (2012). *spa* type t304 was associated with a continuous neonatal ward outbreak in Denmark mainly in 2011 and 2012 (Bartels et al., 2015). Apart from that, it was reported as the dominant strain (39.2%, 31/79) circulating in a tertiary hospital in Oman in 2014 as well as the predominant clone (49.1%, 27/55) in convenience samples collected in Martinique (Uhlemann et al., 2012; Udo et al., 2014). Hence, the rise of *spa* type t304 clone among MRSA in our study may reflect the on-going expansion of this clone and suggest the need for on-going epidemiological surveillance.

MSSA strains with *spa* types t127, t091 and t189 were introduced between the year 2009 and 2010 (Wu et al., 2019; Ghasemzadeh-Moghaddam et al., 2011), and they have been reported as the top 20 most frequent *spa* types among MSSA strains in 25 Europe countries (Grundmann et al., 2014). In this study, s*pa* type t127 was present in both MRSA and MSSA strains, however it was more frequently found in MSSA strains (13.7%, 15/84). Despite the clinical origin, *spa* type t127 was recently reported in processed food in China and animals, indicating the risk of transfer of food-associated and animal-associated MRSA to humans or vice versa (Franco et al., 2011; Mulani et al., 2019). *spa* type t091 has been shown to be the major genotype of MSSA with 16.1% (20/124) isolated from bacteraemia patients in China (Chen et al., 2014; He et al., 2013). However, our study reported that only one strain belonged to *spa* type t091 and this strain was isolated from blood. *spa* type t189 has been reported as the predominant MSSA strain in China and was mostly isolated from wounds (Chen et al., 2014). This is in accordance to their findings as in this study, *spa* type t189 (6.4%, 7/84) was mainly isolated from the pus of MSSA patients.

One MSSA strain belonging to *spa* type t008 was isolated from a 50-year old patient with an abscess. *spa* type t008 is the predominant MSSA strain in United States, and it was reported to have over 80% similarity to the well-known community-acquired MRSA USA300 (Miko et al., 2013). MRSA USA300 is a pandemic hypervirulent, community-acquired, MRSA clone that spread and caused an outbreak in the United States in the year 2004 and 2008 (Chadwick et al., 2013). Fontanilla et al. (2010) indicated that USA300 strains of CA-MSSA can cause outbreaks comparable to USA300 strains of CA-MRSA. Other studies also reported that CA-MRSA particularly USA300 was the most frequent cause of all infections such as severe skin and soft tissue, necrotizing fasciitis and pneumonia reporting to emergency departments in United States (Moran et al., 2006; Otto, 2009).

Despite *spa* type t032 being the most prevalent type in this study, there was no association with any specific clinical manifestation. Similar phenomenon had been reported by a previous study whereby molecular genotypes may be independently associated with clinical characteristics of MRSA (Mccarthy et al., 2015). In contrast to MRSA, *spa* type t091 from MSSA strains were statistically associated with SSTI (*p* = 0.0199) albeit with a small sample size (*n* = 25). Therefore, further validation using larger sample cohort collected from different hospitals in Malaysia is required.

Understanding the pathophysiology of *S. aureus* infections associated with *S. aureus* clonal lineage is a major challenge in the field of infectious disease. Larger sample size is needed to investigate the association of clinical manifestations with a specific *spa* type. Furthermore, the observation of untypable strains which might partially due to the limitation of the study where only a single set of primers and an in-house DNA extraction method used. An alternative set of primers and DNA extraction method using lysostaphin should be included to improve the typing sensitivity in future study (Brignoli et al., 2019; Van-tongeren, Degener & Harmsen, 2011). The reason for the serial rises and fall of specific strain types remains poorly understood and the ability to replace a well-established clone is indeed a concern.

## Conclusion

In summary, *spa* type t032 has replaced the previously predominant *spa* type t037 in 2010 in Malaysia. The results of this study have shown that MRSA and MSSA strains have diverse genetic backgrounds with few dominant clones circulating in the studied hospital.

##  Supplemental Information

10.7717/peerj.11195/supp-1 Supplemental Information 1 GenBank Accession Numbers

## Additional Information and Declarations

### DNA Deposition

The following information was supplied regarding the deposition of DNA sequences: Nucleotide sequences are available at GenBank: MRSA strains: MT783708, MT783709, MT834362, MT783710, MT834363, MT809670, MT809671, MT834364, MT834365, MT834366, MT834432, MT834399, MT834454, MT834451, MT834400, MT834452, MT834453, MT834433, MT834367, MT834434, MT834401, MT834445, MT834449, MT834450, MT834368, MT834402, MT834427, MT834403, MT834446, MT834404, MT834369, MT834370, MT834371, MT834405, MT834428, MT834435, MT834372, MT834373, MT834429, MT834441, MT834406, MT834407, MT834408, MT834409, MT834410, MT834442, MT834411, MT834412, MT834436, MT834413, MT834414, MT834415, MT834416, MT834417, MT834418, MT834437, MT834443, MT834419, MT834420, MT834421, MT834444, MT834430, MT834422, MT834423, MT834424, MT834431, MT834425, MT834438, MT834439 and MT834426. MSSA strains: MT834474, MT834481, MT834447, MT834455, MT834509, MT834375, MT834376, MT834488, MT834475, MT834456, MT834489, MT834377, MT834460, MT834378, MT834379, MT834461, MT834490, MT834457, MT834482, MT834491, MT834380, MT834483, MT834381, MT834382, MT834484, MT834505, MT834506, MT834492, MT834507, MT834485, MT834476, MT834502, MT834486, MT834383, MT834477, MT834503, MT834384, MT834493, MT834494, MT834385, MT834386, MT834462, MT834463, MT834387, MT834388, MT834464, MT834465, MT834513, MT834389, MT834495, MT834466, MT834390, MT834467, MT834468, MT834448, MT834487, MT834511, MT834496, MT834391, MT834497, MT834392, MT834504, MT834469, MT834498, MT834470, MT834393, MT834471, MT834394, MT834395, MT834472, MT834458, MT834479, MT834512, MT834396, MT834499, MT834473, MT834500, MT834397, MT834514, MT834501, MT834459, MT834480, MT834398 and MT834374.

### Data Availability

The following information was supplied regarding data availability: Raw sequencing data are available as Supplementary Files.
