# Supplementary material for: spa diversity of methicillin-resistant and -susceptible Staphylococcus aureus in clinical strains from Malaysia: a high prevalence of invasive European spa-type t032
Source: PeerJ. 2021 Apr 8;9:e11195. doi: 10.7717/peerj.11195 (PMC8038637; doi:10.7717/peerj.11195)
Supplement: Supplemental Information 1 [file peerj-09-11195-s001.docx]

| **Supplementary file:** | **Sequence ID** | **Accession No** | **Lab strain ID** |
| --- | --- | --- | --- |
| 1 | BankIt2366770 Seq1 | MT783708 | R1 |
| 2 | BankIt2366895 Seq2 | MT783709 | R2 |
| 3 | BankIt2370818 Seq3 | MT834362 | R3 |
| 4 | BankIt2367197 Seq4 | MT783710 | R5 |
| 5 | BankIt2370832 Seq5 | MT834363 | R7 |
| 6 | BankIt2367237 Seq6 | MT809670 | R9 |
| 7 | BankIt2367237 Seq7 | MT809671 | R11 |
| 8 | BankIt2370834 Seq8 | MT834364 | R12 |
| 9 | BankIt2370836 Seq9 | MT834365 | R13 |
| 10 | BankIt2370839 Seq10 | MT834366 | R16 |
| 11 | BankIt2370867 Seq11 | MT834432 | R17 |
| 12 | BankIt2370859 Seq12 | MT834399 | R18 |
| 13 | BankIt2371034 Seq13 | MT834454 | R19 |
| 14 | BankIt2370873 Seq14 | MT834451 | R21 |
| 15 | BankIt2370859 Seq15 | MT834400 | R22 |
| 16 | BankIt2370873 Seq16 | MT834452 | R23 |
| 17 | BankIt2370873 Seq17 | MT834453 | R24 |
| 18 | BankIt2370867 Seq18 | MT834433 | R25 |
| 19 | BankIt2370877 Seq19 | MT834367 | R26 |
| 20 | BankIt2370867 Seq20 | MT834434 | R28 |
| 21 | BankIt2370859 Seq21 | MT834401 | R30 |
| 22 | BankIt2370871 Seq22 | MT834445 | R31 |
| 23 | BankIt2370872 Seq23 | MT834449 | R35 |
| 24 | BankIt2370872 Seq24 | MT834450 | R36 |
| 25 | BankIt2371035 Seq25 | MT834368 | R40 |
| 26 | BankIt2370859 Seq26 | MT834402 | R42 |
| 27 | BankIt2370864 Seq27 | MT834427 | R46 |
| 28 | BankIt2370859 Seq28 | MT834403 | R47 |
| 29 | BankIt2370871 Seq29 | MT834446 | R48 |
| 30 | BankIt2370859 Seq30 | MT834404 | R49 |
| 31 | BankIt2371038 Seq31 | MT834369 | R51 |
| 32 | BankIt2371039 Seq32 | MT834370 | R52 |
| 33 | BankIt2371040 Seq33 | MT834371 | R54 |
| 34 | BankIt2370859 Seq34 | MT834405 | R57 |
| 35 | BankIt2370864 Seq35 | MT834428 | R58 |
| 36 | BankIt2370867 Seq36 | MT834435 | R59 |
| 37 | BankIt2371042 Seq37 | MT834372 | R60 |
| 38 | BankIt2371043 Seq38 | MT834373 | R62 |
| 39 | BankIt2370864 Seq39 | MT834429 | R63 |
| 40 | BankIt2370869 Seq40 | MT834441 | R65 |
| 41 | BankIt2370859 Seq41 | MT834406 | R66 |
| 42 | BankIt2370859 Seq42 | MT834407 | R69 |
| 43 | BankIt2370859 Seq43 | MT834408 | R72 |
| 44 | BankIt2370859 Seq44 | MT834409 | R73 |
| 45 | BankIt2370859 Seq45 | MT834410 | R74 |
| 46 | BankIt2370869 Seq46 | MT834442 | R76 |
| 47 | BankIt2370859 Seq47 | MT834411 | R77 |
| 48 | BankIt2370859 Seq48 | MT834412 | R78 |
| 49 | BankIt2370867 Seq49 | MT834436 | R79 |
| 50 | BankIt2370859 Seq50 | MT834413 | R80 |
| 51 | BankIt2370859 Seq51 | MT834414 | R81 |
| 52 | BankIt2370859 Seq52 | MT834415 | R83 |
| 53 | BankIt2370859 Seq53 | MT834416 | R84 |
| 54 | BankIt2370859 Seq54 | MT834417 | R85 |
| 55 | BankIt2370859 Seq55 | MT834418 | R86 |
| 56 | BankIt2370867 Seq56 | MT834437 | R89 |
| 57 | BankIt2370870 Seq57 | MT834443 | R90 |
| 58 | BankIt2370859 Seq58 | MT834419 | R91 |
| 59 | BankIt2370859 Seq59 | MT834420 | R92 |
| 60 | BankIt2370859 Seq60 | MT834421 | R94 |
| 61 | BankIt2370870 Seq61 | MT834444 | R95 |
| 62 | BankIt2370864 Seq62 | MT834430 | R101 |
| 63 | BankIt2370859 Seq63 | MT834422 | R102 |
| 64 | BankIt2370859 Seq64 | MT834423 | R103 |
| 65 | BankIt2370859 Seq65 | MT834424 | R106 |
| 66 | BankIt2370864 Seq66 | MT834431 | R110 |
| 67 | BankIt2370859 Seq67 | MT834425 | R113 |
| 68 | BankIt2370867 Seq68 | MT834438 | R116 |
| 69 | BankIt2370867 Seq69 | MT834439 | R117 |
| 70 | BankIt2370859 Seq70 | MT834426 | R120 |
| 71 | BankIt2371052 Seq71 | MT834474 | S3 |
| 72 | BankIt2371055 Seq72 | MT834481 | S6 |
| 73 | BankIt2370871 Seq73 | MT834447 | S10 |
| 74 | BankIt2371034 Seq74 | MT834455 | S12 |
| 75 | BankIt2371072 Seq75 | MT834509 | S14 |
| 76 | BankIt2371096 Seq76 | MT834375 | S15 |
| 77 | BankIt2371101 Seq77 | MT834376 | S16 |
| 78 | BankIt2371057 Seq78 | MT834488 | S18 |
| 79 | BankIt2371052 Seq79 | MT834475 | S20 |
| 80 | BankIt2371034 Seq80 | MT834456 | S22 |
| 81 | BankIt2371057 Seq81 | MT834489 | S24 |
| 82 | BankIt2371102 Seq82 | MT834377 | S27 |
| 83 | BankIt2371046 Seq83 | MT834460 | S33 |
| 84 | BankIt2371105 Seq84 | MT834378 | S35 |
| 85 | BankIt2371106 Seq85 | MT834379 | S38 |
| 86 | BankIt2371046 Seq86 | MT834461 | S39 |
| 87 | BankIt2371057 Seq87 | MT834490 | S43 |
| 88 | BankIt2371034 Seq88 | MT834457 | S44 |
| 89 | BankIt2371055 Seq89 | MT834482 | S49 |
| 90 | BankIt2371059 Seq90 | MT834491 | S54 |
| 91 | BankIt2371108 Seq91 | MT834380 | S59 |
| 92 | BankIt2371055 Seq92 | MT834483 | S60 |
| 93 | BankIt2371111 Seq93 | MT834381 | S63 |
| 94 | BankIt2371113 Seq94 | MT834382 | S65 |
| 95 | BankIt2371055 Seq95 | MT834484 | S68 |
| 96 | BankIt2371070 Seq96 | MT834505 | S70 |
| 97 | BankIt2371070 Seq97 | MT834506 | S71 |
| 98 | BankIt2371059 Seq98 | MT834492 | S73 |
| 99 | BankIt2371070 Seq99 | MT834507 | S74 |
| 100 | BankIt2371055 Seq100 | MT834485 | S78 |
| 101 | BankIt2371052 Seq101 | MT834476 | S79 |
| 102 | BankIt2371067 Seq102 | MT834502 | S81 |
| 103 | BankIt2371055 Seq103 | MT834486 | S82 |
| 104 | BankIt2371115 Seq104 | MT834383 | S84 |
| 105 | BankIt2371052 Seq105 | MT834477 | S85 |
| 106 | BankIt2371067 Seq106 | MT834503 | S87 |
| 107 | BankIt2371120 Seq107 | MT834384 | S92 |
| 108 | BankIt2371059 Seq108 | MT834493 | S93 |
| 109 | BankIt2371059 Seq109 | MT834494 | S94 |
| 110 | BankIt2371121 Seq110 | MT834385 | S95 |
| 111 | BankIt2371122 Seq111 | MT834386 | S96 |
| 112 | BankIt2371046 Seq112 | MT834462 | S98 |
| 113 | BankIt2371046 Seq113 | MT834463 | S99 |
| 114 | BankIt2371134 Seq114 | MT834387 | S100 |
| 115 | BankIt2371136 Seq115 | MT834388 | S101 |
| 116 | BankIt2371046 Seq116 | MT834464 | S102 |
| 117 | BankIt2371046 Seq117 | MT834465 | S104 |
| 118 | BankIt2371094 Seq118 | MT834513 | S105 |
| 119 | BankIt2371139 Seq119 | MT834389 | S106 |
| 120 | BankIt2371061 Seq120 | MT834495 | S107 |
| 121 | BankIt2371046 Seq121 | MT834466 | S108 |
| 122 | BankIt2371140 Seq122 | MT834390 | S109 |
| 123 | BankIt2371046 Seq123 | MT834467 | S112 |
| 124 | BankIt2371046 Seq124 | MT834468 | S113 |
| 125 | BankIt2370871 Seq125 | MT834448 | S121 |
| 126 | BankIt2371055 Seq126 | MT834487 | S123 |
| 127 | BankIt2371091 Seq127 | MT834511 | S125 |
| 128 | BankIt2371061 Seq128 | MT834496 | S126 |
| 129 | BankIt2371141 Seq129 | MT834391 | S128 |
| 130 | BankIt2371061 Seq130 | MT834497 | S131 |
| 131 | BankIt2371144 Seq131 | MT834392 | S133 |
| 132 | BankIt2371067 Seq132 | MT834504 | S135 |
| 133 | BankIt2371046 Seq133 | MT834469 | S136 |
| 134 | BankIt2371061 Seq134 | MT834498 | S139 |
| 135 | BankIt2371046 Seq135 | MT834470 | S140 |
| 136 | BankIt2371149 Seq136 | MT834393 | S141 |
| 137 | BankIt2371046 Seq137 | MT834471 | S142 |
| 138 | BankIt2371152 Seq138 | MT834394 | S143 |
| 139 | BankIt2371153 Seq139 | MT834395 | S147 |
| 140 | BankIt2371046 Seq140 | MT834472 | S148 |
| 141 | BankIt2371034 Seq141 | MT834458 | S150 |
| 142 | BankIt2371052 Seq142 | MT834479 | S153 |
| 143 | BankIt2371091 Seq143 | MT834512 | S154 |
| 144 | BankIt2371155 Seq144 | MT834396 | S155 |
| 145 | BankIt2371063 Seq145 | MT834499 | S156 |
| 146 | BankIt2371046 Seq146 | MT834473 | S157 |
| 147 | BankIt2371063 Seq147 | MT834500 | S158 |
| 148 | BankIt2371157 Seq148 | MT834397 | S159 |
| 149 | BankIt2371094 Seq149 | MT834514 | S161 |
| 150 | BankIt2371063 Seq150 | MT834501 | S163 |
| 151 | BankIt2371034 Seq151 | MT834459 | S164 |
| 152 | BankIt2371052 Seq152 | MT834480 | S167 |
| 153 | BankIt2371162 Seq153 | MT834398 | S169 |
| 154 | BankIt2371064 Seq154 | MT834374 | S171 |
